# The Effect of Propofol on the Canine Sphincter of Oddi

**DOI:** 10.1155/1994/78764

**Published:** 1994

**Authors:** T. H. Baron, C. B. Dalton, P. B. Cotton, G. R. May, L. G. Milton, R. S. Chari, W. C. Meyers

**Affiliations:** 1 Department of Medicine Division of Gastroenterology Duke University Medical Center Durham N. C. USA; 2 Department of Surgery Duke University Medical Center Durham N. C. USA; 3 Division of Gastroenterology 633 ZRB University Station Birmingham AL 35294 USA

## Abstract

To assess the effect of propofol on the canine sphincter of Oddi (SO), sphincter of Oddi manometry (SOM) was performed in fasting dogs which had undergone cholecystectomy and placement of modified Thomas duodenal cannulae. Using two water-perfused, single-lumen manometric catheters, SO and duodenal pressures were measured simultaneously. Baseline SO activity was recorded for at least one complete interdigestive cycle followed by bolus injections of propofol (Diprivan ^®^) (N = 31) from 0.1 to 4.0 mg/kg during Phase I of the Migrating Motor Complex (MMC).

When propofol was administered in bolus doses ≤ 0.5 mg/kg, no change in SO or duodenal motor function was seen. In doses ≥ 0.5 mg/kg, SO basal pressure, amplitude, and frequency of contractions increased significantly. Increases in duodenal activity paralleled SO activity. Our results suggest that propofol in low doses may be useful for sedation during Sphincter of Oddi manometry in humans. Further studies of the effects of propofol on the human sphincter of Oddi are warranted.


HPB Surgery, 1994, Vol. 7, pp. 297–304
Reprints available directly from the publisher
Photocopying permitted by license only

© 1994 Harwood Academic Publishers GmbH
Printed in the United States of America

### THE EFFECT OF PROPOFOL ON THE CANINE SPHINCTER OF ODDI

T. H. BARON[1]*, C. B. DALTON[1], P. B. COTTON[1], G. R. MAY[1], L. G. MILTON[2], R. S. CHARI[2] and W. C. MEYERS[2]

[1] *Department of Medicine, Division of Gastroenterology*
[2] *Department of Surgery, Duke University Medical Center, Durham, N. C. (U.S.A.)*

To assess the effect of propofol on the canine sphincter of Oddi (SO), sphincter of Oddi manometry (SOM) was performed in fasting dogs which had undergone cholecystectomy and placement of modified Thomas duodenal cannulae. Using two water-perfused, single-lumen manometric catheters, SO and duodenal pressures were measured simultaneously. Baseline SO activity was recorded for at least one complete interdigestive cycle followed by bolus injections of propofol (Diprivan®) (N = 31) from 0.1 to 4.0 mg/kg during Phase I of the Migrating Motor Complex (MMC).

When propofol was administered in bolus doses $\leqslant 0.5$ mg/kg, no change in SO or duodenal motor function was seen. In doses $\geqslant 0.5$ mg/kg, SO basal pressure, amplitude, and frequency of contractions increased significantly. Increases in duodenal activity paralleled SO activity. Our results suggest that propofol in low doses may be useful for sedation during Sphincter of Oddi manometry in humans. Further studies of the effects of propofol on the human sphincter of Oddi are warranted.

KEY WORDS: Propofol, Oddi's sphincter, motility, drug effects, animal, dogs.

### INTRODUCTION

Sphincter of Oddi manometry (SOM) during endoscopic retrograde cholangiopancreatography (ERCP) is increasingly performed in the evaluation of post-cholecystectomy pain[1], allowing identification of a subset of patients with sphincter of Oddi dysfunction who respond to sphincterotomy[2]. Although conscious sedation during diagnostic and therapeutic ERCP is usually achieved with a combination of a benzodiazepine and an opiate, opiate analgesics have been shown to alter SO pressures[3,4] and may prevent accurate measurement of SO pressure. To date, diazepam (Valium®) is the only sedative-hypnotic recommended for conscious sedation during SOM, since it has been demonstrated not to affect SO motility[3,5]. However, many patients are inadequately sedated during SOM using diazepam alone. Thus, the ideal sedative-hypnotic for use during SOM should provide adequate sedation without altering SO pressure or motility.

Propofol (Diprivan®) is a new sedative-hypnotic agent producing dose-dependent depression of the central nervous system similar to that of barbiturates and benzodiazepines[6–8]. Propofol (2, 6 di-isopropylphenol), an alkylphenol, is structurally

* Address correspondence to: Todd H. Baron, M.D, Division of Gastroenterology 633 ZRB, University Station, Birmingham, AL, U.S.A. 35294.

unrelated to all other intravenous anesthetic agents[9]. Initially introduced as an anesthetic induction agent, low-dose propofol has become increasingly popular as a sedative-hypnotic agent for conscious sedation[10–12], administered by either bolus or continuous infusion.

Various animal models have been used to define the SO responses to drugs, hormones, and other regulatory substances. Numerous *in vitro* and *in vivo* studies suggest the SO in dogs functions much like the human SO, both physiologically and pharmacologically[13,14].

The purpose of this study was to determine the effects of propofol at various doses on the motility of the canine SO.

### MATERIALS AND METHODS

### *Animal Preparation*

Six randomly selected dogs weighing 17–23 kg underwent cholecystectomy and placement of modified Thomas duodenal cannulae centered opposite the biliary papilla as previously described[15,16]. The animals were trained to stand quietly during a 6-week recovery period.

### *Equipment*

SO manometry was performed using a Gould transducer and paper recorder (models 13-4615-50 and 2800S, respectively) in conjunction with a pnemo-hydraulic infusion system (Arndorfer Medical Specialities, Greendale, WI). The post-occlusion pressure rise was 200 mm Hg $sec^{-1}$, and paper speed 1 mm $sec^{-1}$. A 5Fr, side-hole catheter (Wilson-Cook Medical®, Winston Salem, U.S.) continuously perfused with de-ionized water at 0.25 ml/min was used for monitoring SO pressure. A 5Fr, end-hole water-perfused catheter (Wilson-Cook) was placed into the duodenal lumen for measuring duodenal pressure. Catheters were secured in postion using a cork in the Thomas cannula (Figure 1).

### *Cannulation Technique and Materials*

A 22-gauge angiocatheter was placed into the radial vein, providing access for intravenous (i.v.) injections. After gently exposing the papilla, the manometry catheter was inserted deeply into the common bile duct. The biliary catheter was withdrawn slowly across the sphincter until the high pressure zone (HPZ) was located by monitoring the pressure tracing and then maintained at a constant position throughout the study.

Ten complete sphincter of Oddi studies were performed in conscious fasting dogs (mean study length 5.3 hours, range 3.8–6.3 hours). Animals were supported in the standing position using a modified Pavlov sling. One complete cycle of the MMC was identified manometrically. All SO measurements (pre- and post-drug) were obtained during Phase I. Sphincter of Oddi measurements included resting sphincter pressure (basal SO pressure minus basal duodenal pressure), as well as amplitude and frequency

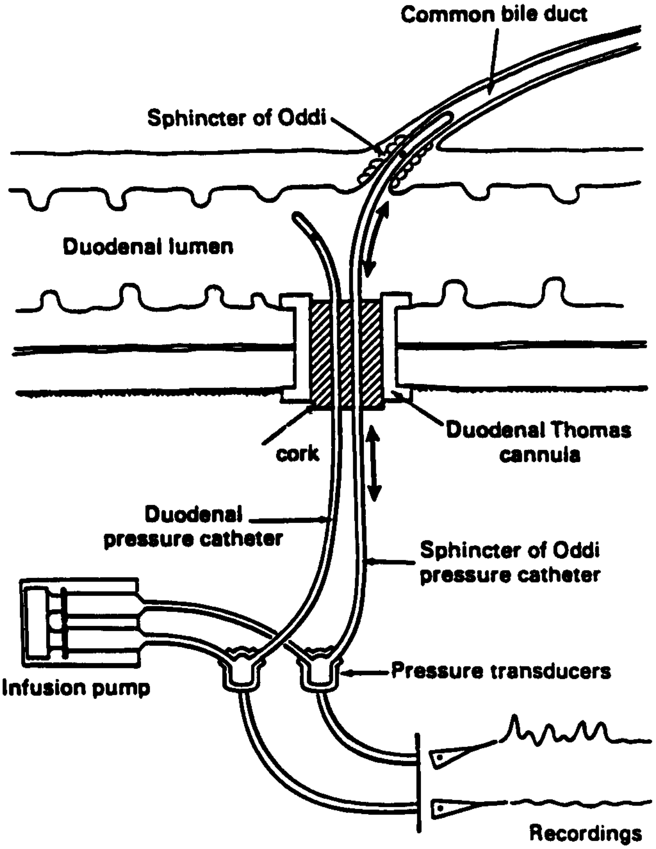

**Figure 1** Manometry system used for simultaneous measurement of biliary sphincter and duodenal pressure. (From: Thompson, J. C., Greely, G. H., Rayford P. L., Townsend, C. M., (1987) Surgical Techniques. In Gastrointestinal Endocrinology, p. 63. New York: McGraw-Hill, Inc., with permission.)

of phasic contractions. Measurements of SO pressure and motility were recorded after injection of CCK-octapeptide, 0.05 $\mu$g/kg in 3 dogs to serve as positive control. Propofol, 10 mg/ml (Diprivan®, ICI Pharmaceuticals, Wilmington, DE), was given by rapid i.v. bolus in incremental doses from 0.1 mg/kg to 4.0 mg/kg.

### *Statistical Methods*

All data are expressed as mean $\pm$ SEM (standard error of the mean). Non-parametric data were analyzed using the Wilcoxon sign rank test. Statistical significance was defined as a probability level, $p < 0.05$.

### RESULTS

In 3 of 6 animals given CCK, SO basal pressure and contractility were reduced as expected. SO basal pressure and motility remained stable throughout the pre-drug study period in all animals. Bolus doses of propofol $< 0.5$ mg/kg (mean dose 0.25 mg/kg, $n = 16$) did not significantly change SO pressure (9.9 $\pm$ 0.6 mm Hg $\rightarrow$ 10.9 $\pm$ 0.6, mean $\pm$ SEM, $p = 0.2$) or alter duodenal activity (Figure 2). However, doses of propofol $\geqslant 0.5$ mg/kg significantly increased SO pressure, with a trend toward dose response. Bolus doses of propofol between 0.5 and 1.0 mg/kg (mean 0.68 mg/kg, $n = 6$) increased SO pressure from 7.8 $\pm$ 1.1 $\rightarrow$ 13.8 $\pm$ 2.4, $p = 0.03$; doses $>$ 1.0 mg/kg (mean 2.3 mg/kg, $n = 9$) increased SO pressure from 6.6 $\pm$ 0.9 $\rightarrow$ 13.8 $\pm$ 1.8, $p = 0.004$ (Figure 2). Bolus doses of propofol $< 0.5$ mg/kg had no effect on amplitude or frequency of contractions, while doses $\geqslant 0.5$ mg/kg increased SO amplitude and frequency (Table 1). Duodenal contractility increased concomitantly with SO contractility following propofol boluses of $\geqslant 0.5$ mg/kg. The duration of propofol's effect on motility was 2–5 minutes when given in doses of 0.5 to 1.0 mg/kg. Figure 3 illustrates the effects

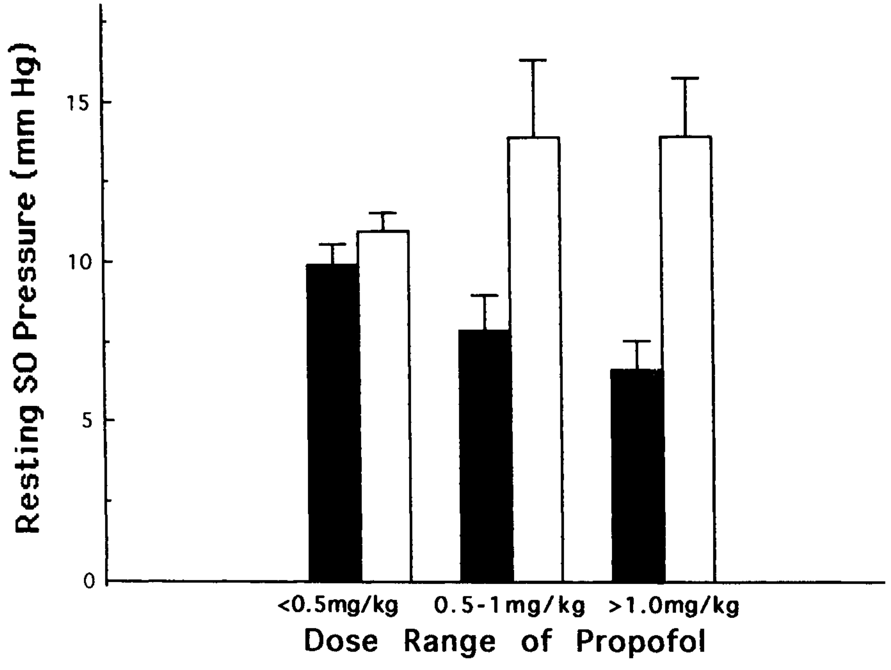

**Figure 2** The effect of propofol on the canine sphincter of Oddi at three dose ranges. Black bars represent the pre-drug sphincter pressure; white bars represent post-drug sphincter pressure (mean $\pm$ SEM).

**Table 1** Effect of propofol on amplitude and frequency of SO contractions

| *Propofol* (mg/kg) | *Amp (pre)* | *Amp (post)* | *Freq (pre)* | *Freq (post)* |
|---|---|---|---|---|
| 0.1 – 0.45 | 9.8 $\pm$ 1.1 | 10.7 $\pm$ 1.4 | 10.7 $\pm$ 0.9 | 12.2 $\pm$ 1.0 |
| 0.5 – 1.0 | 9.5 $\pm$ 0.9 | 17.3 $\pm$ 2.6* | 8.8 $\pm$ 1.1 | 13.8 $\pm$ 1.9 |
| > 1.0 | 10.6 $\pm$ 1.0 | 24.9 $\pm$ 3.4* | 9.0 $\pm$ 0.7 | 15.4 $\pm$ 1.0* |

Amp = amplitude (mm Hg); Freq = frequency ($sec^{-1}$)* $p < 0.05$.

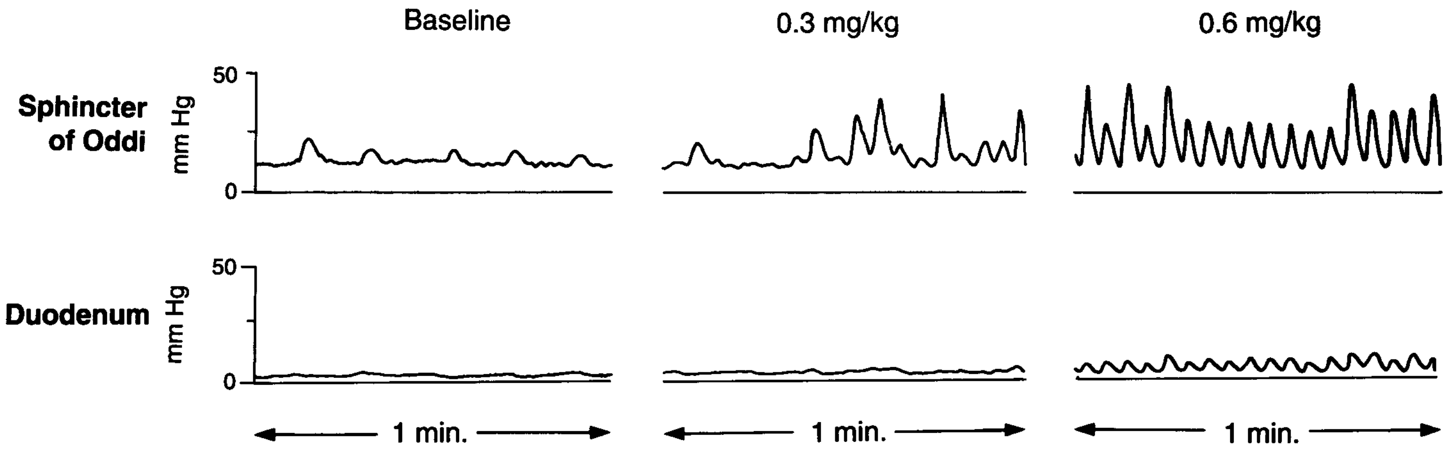

**Figure 3** Representative tracings of sphincter of Oddi and duodenal pressure during baseline and maximal drug effect.

of low-dose propofol on the canine sphincter. At doses > 1 mg/kg duodenal and SO contractions resembled those seen during Phase III of the MMC.

### DISCUSSION

The effect of propofol on sphincter of Oddi motility is unknown. We have demonstrated that low-dose propofol in bolus doses < 0.5 mg/kg have no effect on sphincter of Oddi or duodenal motility in the canine. Adequate conscious sedation in humans can be achieved with intermittent propofol boluses of less than 0.5 mg/kg (personal communication, P.S.A. Glass, M.D., 1993). Although the canine is less sensitive to the sedative effects of propofol than humans[17], we observed the animals to be sedated when repetitive bolus doses of < 0.5 mg/kg were given. Due to the redistribution characteristics of the drug, cumulative effects are not seen after multiple bolus doses or prolonged infusion.

Rosa and colleagues[18] administered repeated, small boluses of propofol (0.6 mg/kg followed by 0.3 mg/kg) during regional anesthesia. Excellent conscious sedation was achieved without effect on central respiratory drive, gas exchange, or respiratory pattern. Advantages of propofol include a rapid onset of action and a large volume of distribution leading to rapid recovery[9]. In addition, it possesses favorable amnestic, analgesic, and anti-emetic properties[19,20]. Phlebitis occurs in less than 1% of patients[21].

Propofol has been used for conscious sedation during gastrointestinal (G.I.) procedures with significantly faster recovery times than diazepam or midazolam[22–25]. Dubois *et al.*[23] used propofol by continuous infusion at a mean dose of 4.3 mg/kg/hr for conscious sedation during 100 G.I. procedures (including ERCP and sphincterotomy).

In this study, higher doses of propofol significantly increased SO contractility. Since the canine SO is entirely within the intramural segment of the duodenum[26], it is possible that SO motility was increased secondary to an increase in duodenal contractility. These changes in motility may be explained by several mechanisms. Propofol causes vasodilation when administered in doses required for induction and maintenance of general anesthesia[12]. Using endothelial cells *in vitro*, Xuan *et al.*[27] demonstrated that propofol interferes with intracellular calcium mobilization which may be the mechanism for propofol induced relaxation of endothelial smooth muscle. However, intracellular calcium release plays a minor role in gastrointestinal smooth muscle contraction[28]. Propofol may shift extracellular calcium into cells, increasing contractility. Additionally, sub-hypnotic doses of propofol alleviate pruritus in cholestatic liver disease[29], possibly via interaction with opioid receptors in the central nervous system. Since opiates cause alterations in the contractility of the SO, higher doses of propofol may alter SO contractility through interaction with opioid receptors.

We feel propofol possesses many advantages over diazepam for conscious sedation, including analgesic properties and rapid recovery. Studies in patients with and without SO dysfunction are needed to determine if propofol (alone and in combination with diazepam) has clinically significant effects on the human SO. We have demonstrated that propofol doses of 0.5 to 1.0 mg/kg briefly affect the canine SO, consistent with the drug's rapid redistribution and short half-life. Thus, even if demonstrated to affect the human SO, propofol may be useful for sedation during SO manometry. Patients not adequately sedated with diazepam alone could be given small bolus doses of propofol until successful cannulation is achieved. An interval of several minutes prior to measuring SO pressures should allow the effects of propofol on the SO to have resolved.

### *Acknowledgements*

The authors wish to thank E. Bozymski, M.D., O. Akwari, M.D., and Wilson-Cook Medical, Inc. for supplying equipment; G. Branum, M.D. for animal preparation; P. S. A. Glass, M.D. for consultation; M. Dyer and S. Bridges for technical support.

### *References*

1. Lans, J. L., Parikh, N. P., Geenen, J. E. (1991) Application of sphincter of oddi manometry in routine clinical investigations. *Endoscopy*, **23**, 139–43
2. Geenen, J. E., Hogan, W. J., Dodds, W. J., Toouli, J., Venu, R. P. (1989) The efficacy of endoscopic sphincterotomy after cholecystectomy in patients with sphincter of oddi dysfunction. *N. Engl. J. Med.*, **320**, 82–7
3. Staritz, M. (1988) Pharmacology of the sphincter of oddi. *Endoscopy*, **20**, 171–4
4. Staritz, M., Poralla, T., Manns, M., Zum Buschenfelde, K-HM. (1986) Effect of modern analgesic drugs (Tramadol, Pentacozine, Buprenorphine) on the bile duct sphincter. *Gut*, **27**, 567–9
5. Nebel, O. T. (1975) Manometric evaluation of the papilla of vater. *Gastrointest Endosc.*, **21**, 126–8
6. Doze, V. A., Westphal, L. M., White, P. F. (1986) Comparison of propofol with methohexital for outpatient Anesthesia. *Anesth. Analg.*, **65**, 1189–95
7. Purcell-Jones, G., Yates, A., Baker, J. R. James, I. G. (1987) Comparison of the induction characteristics of thiopental and propofol in children. *Br. J. Anaesth.*, **59**, 1431–6
8. Fragen, R. J. (1988) Diprivan (Propofol): A historical perspective. *Semin. Anesth.*, *VII*, (suppl 1): 1–3
9. Ferrari, L. R., Donlan, J. V. (1992) A comparison of propofol, midazolam, and methohexital for sedation during retrobulbar and peribulbar block. *J. Clin. Anesth.*, **4**, 93–6
10. White, P. F., Negus, J. B. (1991) Sedative infusions during local and regional anesthesia: A comparison of propofol and midazolam. *J. Clin. Anesth.*, **3**, 32–9
11. Valtonen, M., Salonen, M., Forssell, H., Scheinen, M., Viinamaki, O. (1989) Propofol infusion for sedation in outpatient oral surgery, a comparison with diazepam. *Anaesthesia.*, **44**, 730–4
12. Larijani, G. E., Gratz, I., Afshar, M., Jacobi, A. G. (1989) Clinical pharmacology of propofol: an intravenous anesthetic agent. *DICP*, **23**, 743–9
13. Aldman, G. (1988) Some comparative aspects on biliary motility in animal species. *Scand. J. Gastroenterol*, **152**, (Suppl) 63–6
14. Funch-Jensen, P. (1990) Acta. Chirurgica. Scand. Sphinter of odd motility (suppl 553) 1–35
15. Jones, R. S., Yee, T. K., Michielsen, C. E. (1971) A modified Thomas cannula for gastric and intestinal fistulas. *J. Appl. Physiol.*, **30**, 427–8
16. Sievert, C. E. Jr., Potter, T. J., Levine, A. S., Morley, J. E., Silvis, S. E., Vennes, J. A. (1988) Effect of bombesin and gastrin-releasing peptide on canine sphincter of Oddi. *Am. J. Physiol.*, **254**, G361–65
17. Watney, G. C., Pablo L. S. (1992) Median effective dosage of propofol for the induction of anesthesia in dogs. *Am. J. Vet. Res.*, **53**, 2320–2
18. Rosa, G., Conti, G., Oorsi, P., D'Alessandro, F., Di Giugno G., Gasparetto, A. (1992) Effects of low-dose propofol administration on central respiratory drive, gas exchanges, and respiratory pattern. *Acta. Anaesthiol. Scand.*, **36**, 128–31
19. White, P. F. (1988) Propofol: pharmacokinetics and pharmacodynamics. *Semin. Anesth VII*, (suppl 1): 15–17
20. McCollum, J. S. C., Milligan, K. R., Duntel, J. W. (1988) The antiemetic action of propofol. *Anaesthesia*, **43**, 239–40
21. Sebel, P. S., Lowdon, J. D. (1989) Propofol: A new intravenous anesthetic. *Anesthesiology*, **71**, 260–277

22. Gepts, E., Claeys, M. A., Camu, F. Smekens, L. (1985) Infusion of propofol (diprivan) as a sedative technique for colonoscopies. *Postgrad. Med. Journal*, **61**, (Suppl 3) 120–6
23. Dubois, A., Balatoni, E., Peeters, L. P., Baudoux, M. (1988) Use of propofol for sedation during gastrointestinal endoscopies. *Anaesthesia*, **43**, (Suppl) 75–80
24. Patterson, K. W., Casey, P. B., Murray, J. P., O'Boyle C. A., Cunningham, A. J. (1991) Propofol sedation for outpatient upper gastrointestinal endoscopy: comparison with midazolam. *Br. J. Anaesthesia*, **67**, 108–11
25. Church, J. A., Stenton, P. D., Kenney, G. N. C., Anderson, J. R. (1991) Propofol for sedation during endoscopy: assessment of a computer-controlled infusion system. *Gastrointest. Endosc.*, **37**, 175–9
26. Hauge, C. W., Mark, J. B. D. (1965) Common bile duct motility and sphincter mechanism: I. pressure measurements with triple lumen catheter in dogs. *Ann. Surg.*, **162**, 1028–38
27. Xuan, Y., Zhang, J., Glass, P. (1992) Propofol inhibition of endothelin-mediated formation of inositol phosphates in smooth muscle. *Anesthesiology*, **77**, A597
28. Mangel, A. W., Taylor, I. L. (1992) Modulation of electrical activity in gastrointestinal smooth muscle by peptides. *Regulatory Peptide*, **42**, 1–13
29. Borgeat, A., Wilder-Smith. O. H. G., Mentha, G. (1993) Subhypnotic doses of propofol relieve pruritus associated with chronic liver disease. *Gastroentrol*, **104**, 244–7

